# Supplementary material for: Skyrmions in synthetic antiferromagnets and their nucleation via electrical current and ultra-fast laser illumination
Source: Nat Commun. 2022 Aug 16;13:4807. doi: 10.1038/s41467-022-32525-4 (PMC9381802; doi:10.1038/s41467-022-32525-4)
Supplement: Supplementary file 1 — Supplementary information [file 41467_2022_32525_MOESM1_ESM.pdf]

SUPPLEMENTARY INFORMATION

Skyrmions in synthetic antiferromagnets and their nucleation via electrical current and ultra-fast laser illumination

Roméo Juge,[1,*] Naveen Sisodia,[1,*] Joseba Urrestarazu Larrañaga,[1] Qiang Zhang,[1] Van Tuong Pham,[1] Kumari Gaurav Rana,[1] Brice Sarpi,[2] Nicolas Mille,[2] Stefan Stanescu,[2] Rachid Belkhou,[2] Mohamad-Assaad Mawass,[3] Nina Novakovic-Marinkovic,[3] Florian Kronast,[3] Markus Weigand,[3] Joachim Gräfe,[4] Sebastian Wintz,[4] Simone Finizio,[5] Jörg Raabe,[5] Lucia Aballe,[6] Michael Foerster,[6] Mohamed Belmeguenai,[7] Liliana D. Buda-Prejbeanu,[1] Johan Pelloux-Prayer,[1] Justin M. Shaw,[8] Hans T. Nembach,[8,9] Laurent Ranno,[10] Gilles Gaudin,[1] and Olivier Boulle[1,†]

[1] *Univ. Grenoble Alpes, CEA, CNRS, Grenoble INP, IRIG-SPINTEC, 38054 Grenoble, France*

[2] *Synchrotron SOLEIL, L'Orme des Merisiers, 91190 Saint-Aubin, France*

[3] *Helmholtz-Zentrum Berlin für Materialien und Energie, Albert-Einstein-Straße 15, 12489 Berlin, Germany*

[4] *Max Planck Institute for Intelligent Systems, Heisenbergstraße 3, 70569 Stuttgart, Germany*

[5] *Swiss Light Source, Paul Scherrer Institut, 5232 Villigen, Switzerland*

[6] *ALBA Synchrotron Light Facility, 08290 Cerdanyola del Vallès, Barcelona, Spain*

[7] *Laboratoire des Sciences des Procedés et des Matériaux, CNRS, Univ. Paris 13, 93430 Villetaneuse, France*

[8] *Quantum Electromagnetics Division, National Institute of Standards and Technology, Boulder, CO 80309, USA*

[9] *Department of Physics, University of Colorado, Boulder, CO 80309, USA*

[10] *Univ. Grenoble Alpes, CNRS, Institut Néel, 38042 Grenoble, France*

* These authors contributed equally to this work.

† e-mail: olivier.boulle@cea.fr

S1. PREPARATION AND CHARACTERISATION OF THE SAF MULTILAYERS

Three compensated SAF multilayers with slightly different compositions are discussed in the main text, as detailed in the Methods section: (i) **SAF1**, composed of Pt(2.5)/[Pt(0.5)/Co(1.35)/Ru(0.85)/Pt(0.5)/Co(0.3)/$Ni_{80}Fe_{20}$(1.45)/Co(0.3)/Ru(0.85)$]_2$/Pt(2) (thicknesses in nanometres), was used for the observation of SAF skyrmions at zero external magnetic field by STXM (Fig. 1, main text); (ii) **SAF2**, composed of Ta(3)/Pt(3)/[Pt(0.5)/Co(0.2)/$Ni_{80}Fe_{20}$(0.95)/Co(0.2)/Ru(0.85)/Pt(0.5)/Co(1.35)/Ru(0.85)$]_{12}$/Pt(2), was used for the current-induced skyrmion nucleation and annihilation (Fig. 4, main text); (iii) **SAF3**, composed of Ta(3)/Pt(2.25)/[Pt(0.75)/Co(1.49)/Ru(0.85)$]_6$/Pt(1.2) was used for the laser-induced skyrmion nucleation experiments, observed by XMCD-PEEM (Fig. 5, main text).

S1.1. SAF1 multilayer: [Pt/Co/Ru/Pt/Co/NiFe/Co/Ru$]_2$

S1.1.1. Material optimisation

We discuss in this section the preparation and characterisation of SAF1 (Fig. 1 in the main text). It is composed of Pt/Co/NiFe/Co and Pt/Co layers antiferromagnetically coupled through a thin Ru layer via RKKY-type interlayer exchange coupling: [Pt(0.5)/FM1/Ru(0.85)/Pt(0.5)/FM2/ Ru(0.85)$]_2$ where FM1 = Co(0.3)/$Ni_{80}Fe_{20}$(t_{NiFe})/Co(0.3) and FM2 = Co(t_{Co}) (thicknesses in nanometres). The material stacks for the two FM layers, Pt/FM2/Ru and Pt/FM1/Ru, are represented in Fig. S1a and S1b, respectively. In Fig. S1c the material stack for the SAF with $t_{\mathrm{Co}} = 1.35$ nm is represented. For each sample, the buffer layer (Buff.) consists of Ta(3)/Pt(2.5). Fig. S1d-f show the out-of-plane (OOP) hysteresis loops measured by polar magneto-optical Kerr effect (pMOKE) in these three samples. In Fig. S1d, t_{Co} is varied between 1.25 nm and 1.40 nm on different samples and the loops display the gradual transition from perpendicular magnetic anisotropy (PMA) to in-plane (IP) magnetic anisotropy (spin reorientation transition). In Fig. S1e, the $Ni_{80}Fe_{20}$ layer (hereafter NiFe) is deposited as a wedge with $t_{\mathrm{NiFe}} = 0.76 - 1.55$ nm. Here also, the loops display the gradual transition from PMA to IP anisotropy and, for $t_{\mathrm{NiFe}} = 1.32$ nm, a reversal characteristic of a multi-domain state is observed.

Fig. S1f shows the loops for the SAF with $t_{\mathrm{Co}} = 1.35$ nm at different locations on the NiFe wedge. These loops exhibit two reversals, characteristic of antiferromagnetic (AF) coupling of perpendicularly magnetised layers. The amplitude of the central hysteresis, clearly visible for $t_{\mathrm{NiFe}} \leq 1.10$ nm, is proportional to the net magnetic moment. Upon increasing the NiFe thickness,

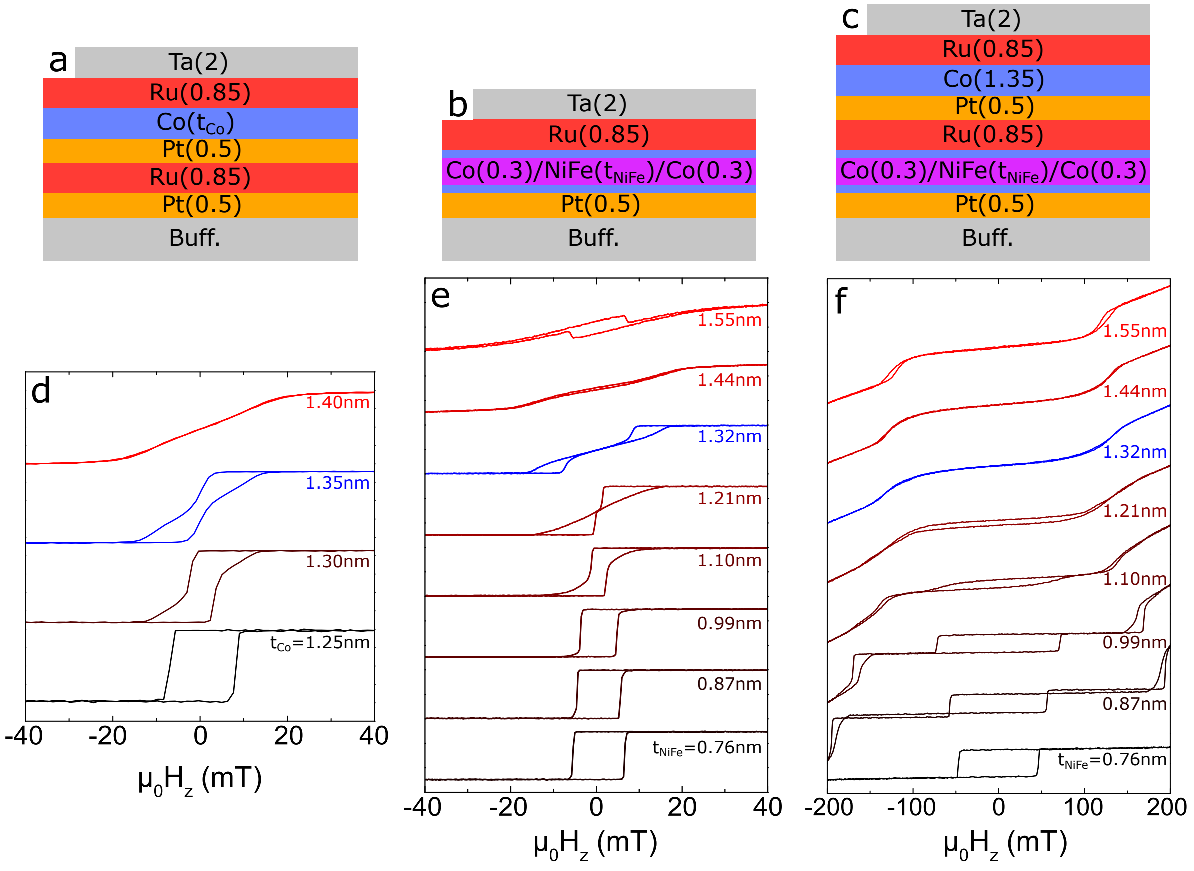

FIG. S1. **Optimisation of SAF1. a, b** Material stacks for the constituent FM layers, (**a**) Pt/FM2/Ru and (**b**) Pt/FM1/Ru, with FM2 = Co(t_{Co}) and FM1 = Co(0.3)/$Ni_{80}Fe_{20}$(t_{NiFe})/Co(0.3) (thicknesses in nanometres). **c** Material stack for the SAF, Pt/FM1/Ru/Pt/FM2/Ru, with t_{Co} = 1.35 nm. Buff. denotes Ta(3)/Pt(2.5). The NiFe layer in (**b**) and (**c**) is deposited as a wedge. **d-f** OOP MOKE hysteresis loops measured in the stacks showed above. The loops in (**d**) correspond to different (non-wedged) samples. In (**e**) and (**f**), each loop is measured at positions 10 mm apart on the wedge.

this amplitude decreases and eventually vanishes for $t_{NiFe} \approx 1.4$ nm, indicating magnetic moment compensation. This is accompanied by a widening of the central hysteresis, consistently with the diminution of the net magnetic moment. Note that the thickness of the Co layers surrounding the NiFe (0.3 nm) was chosen so that the spin reorientation transition of FM1 matches that of FM2. It also allows to increase the magnetisation of FM1 to match that of FM2, since M_s (NiFe) < M_s (Co). The second reversal is followed by a constant-susceptibility region, corresponding to spin-flop processes, characteristic of SAFs with weak anisotropy [1, 2]. Note that the OOP saturation is not reached due to the limited applicable field in these experiments. The field at which this spin-flop transition occurs decreases with increasing NiFe thickness, as a result of the weakening of the anisotropy and of the RKKY coupling [1].

The composition of SAF1 presented in the main text corresponds to $t_{\mathrm{Co}} = 1.35$ nm and $t_{\mathrm{NiFe}} = 1.45$ nm. The magnetic moment of the different components of SAF1 was measured by SQUID magnetometry: a magnetic moment per unit area of $(2.10 \pm 0.07) \times 10^{-3}$ A is measured in Ta(3)/Pt(3)/Co(1.35)/Ru(0.85)/Pt(2) while a moment of $(1.99 \pm 0.07) \times 10^{-3}$ A is measured in Ta(3)/Pt(3)/Co(0.3)/$Ni_{80}Fe_{20}$(1.45)/Co(0.3)/Ru(0.85)/Pt(2), hence confirming that the SAF is compensated (within measurement error).

S1.1.2. Measurement of the Dzyaloshinskii-Moriya interaction by Brillouin light scattering spectroscopy

Brillouin light scattering (BLS) spectroscopy experiments were carried out to extract the Dzyaloshinskii-Moriya interaction (DMI) of the Pt/Co/Ru and Pt/Co/NiFe/Co/Ru multilayers composing SAF1. The principle of the measurement is the following [3, 4]: the magnetisation is saturated in the film plane by an external magnetic field and spin waves (SW) propagating along the direction perpendicular to this field are probed by a laser with a well-defined wave vector k (Damon-Eshbach geometry). The DMI introduces a preferred chirality and leads to an energy difference between SW propagating with opposite wave vectors. This energy difference corresponds to a shift in frequency: $\Delta f(k) = f_{\mathrm{S}}(k) - f_{\mathrm{AS}}(k)$ where f_{S} and f_{AS} are the Stokes (a SW is created) and anti-Stokes (a SW is absorbed) frequencies, respectively. This frequency shift is directly related to the DMI constant D via [3]: $\Delta f(k) = 2\gamma k D/(\pi M_{\mathrm{s}})$ with $\gamma = g\mu_{\mathrm{B}}/\hbar$ the gyromagnetic ratio, M_{s} the saturation magnetisation, g the Landé factor. The DMI constant D can be extracted from the frequency-shift measured for both field polarities or from the slope of Δf for different k. To characterize the DMI in SAF1, we measured it in the Pt/Co/Ru and Pt/Co/NiFe/Co/Ru stacks composing the SAF.

a. DMI in Pt/Co/Ru. The DMI was measured in two samples with a Co thickness (t_{Co}) slightly above (1.5 nm) and below (1.05 nm) that of the actual sample (1.35 nm), with composition Ta(3)/Pt(3)/Ru(0.85)/Pt(0.5)/Co(t_{Co})/Ru(0.85)/Pt(2) and deposited by magnetron sputtering on Si(100) substrates. Fig. S2a shows Δf as a function of the wave vector k for the sample with $t_{\mathrm{Co}} = 1.5$ nm. The negative slope of Δf vs k implies a negative DMI constant, corresponding to a left-handed (or anti-clockwise) chirality, in agreement with the sign of the DMI at the Pt/Co interface [5–7]. From a linear fit of Δf vs k, we extract $|D_{\mathrm{Co1.5}}| = 0.62 \pm 0.04$ mJ m^{-2}. Here we used $g = 2.21$, as measured by ferromagnetic resonance in Pt/Co/MgO [5, 6], and $M_{\mathrm{s}} = 1.43 \pm 0.05$ MA m^{-1}, as was measured from the dependence of the magnetic moment on

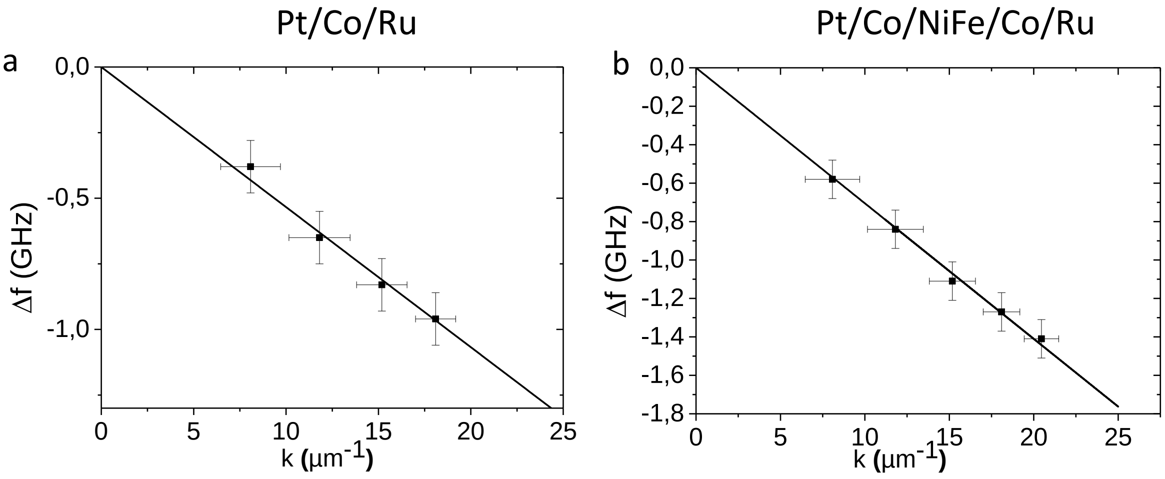

FIG. S2. **Brillouin light scattering measurements in SAF1.** Frequency shift as a function of the spin wave vector measured in **a** Ta(3)/Pt(3)/Ru(0.85)/Pt(0.5)/Co(1.5)/Ru(0.85)/Pt(2) and **b** Ta(3)/Pt(3)/Co(0.3)/NiFe(1.45)/Co(0.3)/Ru(0.85)/Pt(2) (thicknesses in nanometres). The lines are linear fits assuming a zero intercept. The error bars stand for the uncertainty due to the measurement resolution.

the FM film thickness in Pt/Co/Ru thin films [8]. For the sample with $t_{\mathrm{Co}} = 1.05$ nm, we find a frequency shift $\Delta f = (6.22 \pm 0.20) \times 10^{-1}$ GHz at an IP wave vector $k_{\mathrm{x}} = 16.7\ \mu\mathrm{m}^{-1}$ for both field polarities. This yields $|D_{\mathrm{Co1.05}}| = 0.86 \pm 0.06$ mJ m^{-2}. Assuming that the DMI scales as $1/t$, t being the FM thickness [3, 9], a DMI $|D_{\mathrm{Co1.35}}| = 0.67 \pm 0.04$ mJ m^{-2} is estimated for $t_{\mathrm{Co}} = 1.35$ nm.

b. DMI in Pt/Co/NiFe/Co/Ru. The DMI was measured in a sample with a composition close to the Pt/Co/NiFe/Co/Ru layer in SAF1: Ta(3)/Pt(3)/Co(0.3)/NiFe(1.45)/Co(0.3)/Ru(0.85)/Pt(2), deposited by magnetron sputtering on a Si(100) substrate. Fig. S2b shows Δf as a function of the wave vector k measured by BLS. From a linear fit of, we find $|D| = 0.57 \pm 0.11$ mJ m^{-2}. Here we used an average Landé factor of $g = 2.13$ using $g = 2.1$ for the NiFe layer [4] and $g = 2.21$ for the Co layer [5, 6]. An effective saturation magnetisation $M_{\mathrm{s}} = 0.97 \pm 0.15$ MA m^{-1} was calculated by dividing the surface magnetic moment measured by SQUID magnetometry by the total FM thickness.

S1.1.3. Micromagnetic simulations

The simulations were carried out with Mumax3 [10] using the parameters given in Table I measured in the two constituting layers of the SAF (see previous sections).

Using these parameters, we calculated a hysteresis loop on a SAF with lateral dimensions

	Co/NiFe/Co	Co
t_{eff} (nm)	2.05	1.3916
M_{s} (MA m^{-1})	0.9707	1.43
K_{u} (MJ m^{-3})	0.6114	1.3206
$\|D\|$ (mJ m^{-2})	0.57	0.67
A (pJ m^{-1})	6	16
α	0.08	0.12

TABLE I. **Micromagnetic parameters for SAF1.** Effective FM thickness, saturation magnetisation, uniaxial anisotropy constant, DMI constant, exchange constant and magnetic damping.

512×512 nm^2 with periodic boundary conditions along both x and y. To obtain results in a reasonable time frame, a size of 4×4 nm^2 was considered. The simulated hysteresis is shown in Fig. S3, and it reproduces well the experimental VSM loop (Fig. 1b in the main text).

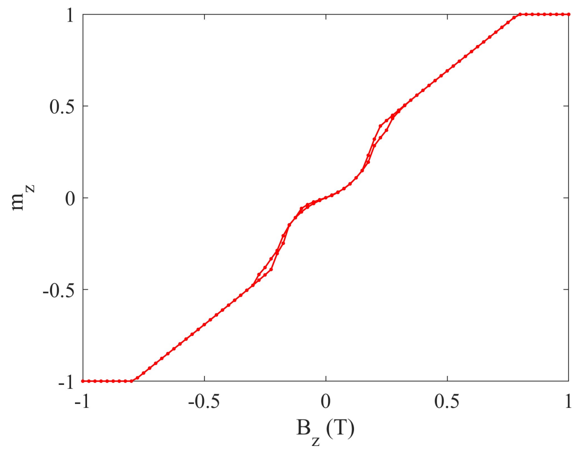

FIG. S3. **Simulated hysteresis loop.** Micromagnetic simulation obtained for the parameters given in Table I.

S1.2. SAF2 multilayer: $[Pt/Co/Ru/Pt/Co/NiFe/Co/Ru]_{12}$

S1.2.1. Material optimisation

We discuss in this section the preparation and characterisation of the SAF2 multilayer used for the current-induced skyrmion nucleation/annihilation experiments (Fig. 4 in the main text). It is composed of Pt/Co/NiFe/Co and Pt/Co layers AF coupled through a thin Ru layer via RKKY-type interlayer exchange coupling: Ta(3)/Pt(2.5)/[Pt(0.5)/FM1/Ru(0.85)/Pt(0.5)/FM2/ $\mathrm{Ru}(0.85)]_{12}$/Pt(2) where FM1 = Co(0.2)/$Ni_{80}Fe_{20}$(0.95)/Co(0.2) and FM2 = Co(0.9) (thicknesses in nanometres). The material stacks for the two constituent FM layers and for the SAF bilayer are shown in Fig. S4a, S4b and S4c, respectively. Fig. S4d-f display the OOP hysteresis loops measured by pMOKE in these three samples. Here, Pt/FM2/Ru exhibits full remanence due to the smaller Co thickness (0.9 nm), while Pt/FM1/Ru is kept close to the spin reorientation transition by reducing the thickness of the Co layers in contact with the NiFe from 0.3 to 0.2 nm. Fig. S4f shows the loop for the SAF bilayer ($N = 1$); it reveals that the magnetisation reversals are sharper, owing to the larger anisotropy. The vibrating sample magnetometry (VSM) loop (Fig. S4h, $N = 1$), which provides a measurement of the total moment, indicates that the SAF is compensated.

For the STXM experiments, the stack of Fig. S4g with $N = 12$ was deposited on both a Si substrate (reference sample) and on a 200-nm-thick 100×100 µm^2 Si_3N_4 membranes on which the track and injection devices were patterned. The OOP hysteresis loops measured by VSM for different N on references samples are shown in Fig. S4h, wherein the signal is expressed in units of the total number of constituent FM layers ($2N$). These loops exhibit a vanishing magnetic moment and zero susceptibility to the field in the central plateau region, which is characteristic of uniformly magnetised, compensated SAFs. For $N = 1$, only one reversal exists, which defines the interlayer exchange field $\mu_0|H_{\mathrm{RKKY}}| \approx 200$ mT. For $N > 1$, an additional reversal appears around $|H_z| = 2H_{\mathrm{RKKY}}$. It corresponds to the reversal of the internal FM layers, i.e., all but the top-most and the bottom-most FM layer: since each internal FM layer is AF coupled on both sides (below and above), twice the external field is required to switch their magnetisation [11]. This can be seen from the amplitude of the different plateaus in Fig. S4h: for $H_{\mathrm{RKKY}} < |H_z| < 2H_{\mathrm{RKKY}}$, the net magnetisation is $|m_z| = 2$, independently of N, while $|m_z| = 2N$ at saturation ($|H_z| \gg 2H_{\mathrm{RKKY}}$). This means that $(N - 1)$ layers have switched between the two plateaus.

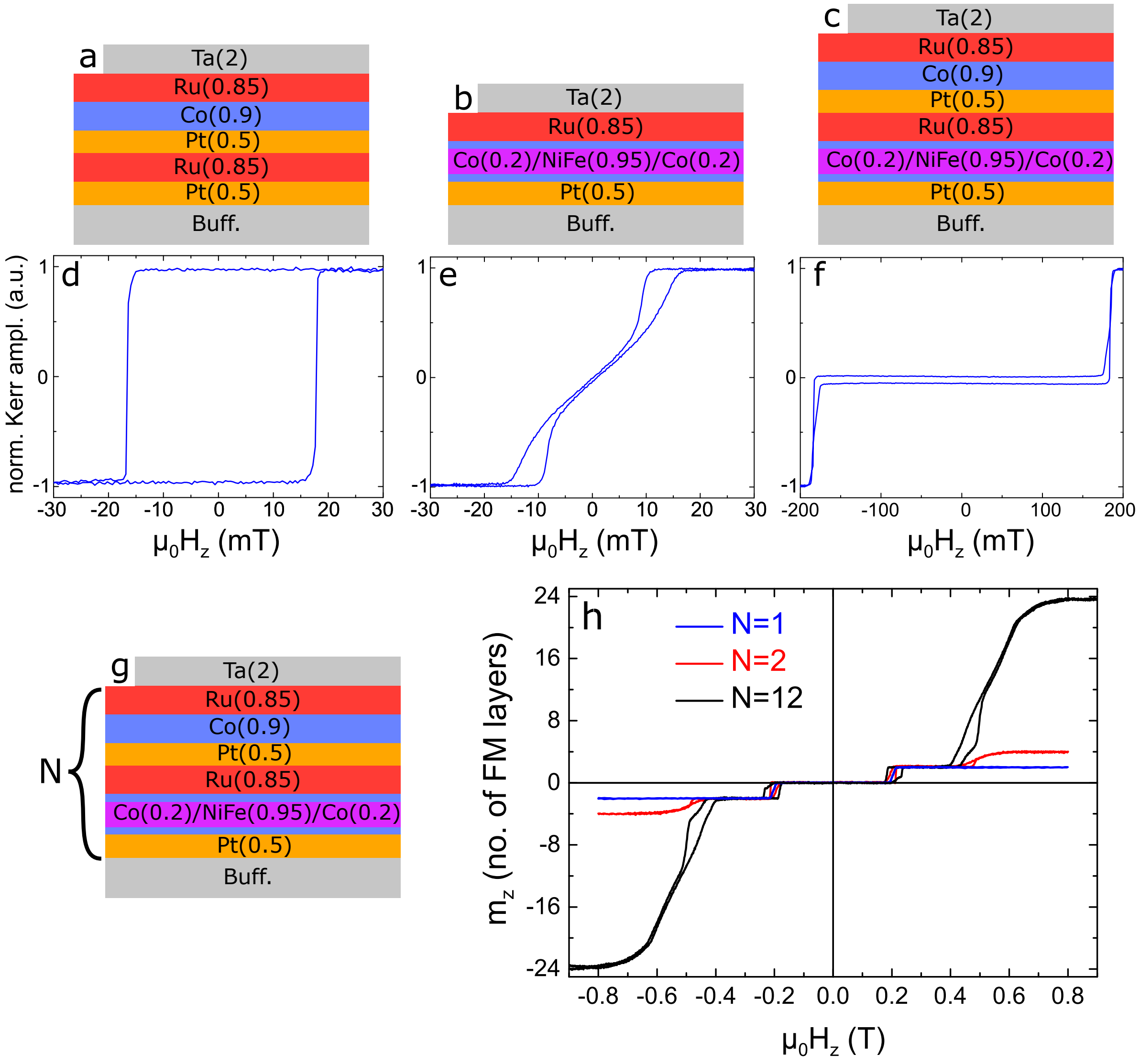

FIG. S4. **Optimisation of SAF2.** **a, b** Material stacks for the constituent FM layers, (**a**) Pt/FM2/Ru and (**b**) Pt/FM1/Ru, with FM2 = Co(0.9) and FM1 = Co(0.2)/$Ni_{80}Fe_{20}$(0.95)/Co(0.2) (thicknesses in nanometres). **c** Material stack for the SAF, Pt/FM1/Ru/Pt/FM2/Ru. Buff. denotes Ta(3)/Pt(2.5). **d-f** OOP MOKE hysteresis loops measured in the stacks showed above. **g** Material stack for the multi-layered SAF. **h** OOP VSM hysteresis loops measured for different N. The signal is normalised and multiplied by the total number of constituent FM layers ($2N$).

S1.2.2. Measurement of the Dzyaloshinskii-Moriya interaction by Brillouin light scattering spectroscopy

To characterize the DMI in SAF2, we performed BLS measurements in Pt/Co/Ru and in Pt/Co/NiFe/Co/Ru stacks with composition close to that of SAF2 and deposited by magnetron sputtering on Si(100) wafers.

a. DMI in Pt/Co/Ru. The DMI in the Pt/Co(0.9)/Ru layer was estimated from the DMI measurement in Ta(3)/Pt(3)/Ru(0.85)/Pt(0.5)/Co(1.05)/Ru(0.85)/Pt(2) presented in section S1.1.2.a. Assuming the DMI scales linearly as $1/t$, a DMI value $|D| = 1.00 \pm 0.07$ mJ m^{-2} is estimated.

b. DMI in Pt/Co/NiFe/Co. The DMI was measured in a sample with the same composition as in SAF2: Ta(3)/Pt(3)/Ru(0.85)/Pt(0.5)/Co(0.2)/NiFe(0.96)/Co(0.2)/Ru(0.85)/Pt(2), deposited by magnetron sputtering on a Si(100) substrate. A frequency shift $\Delta f = (4.35 \pm 0.18) \times 10^{-1}$ GHz is measured at an IP wave vector $k = 16.7$ µm^{-1}. This leads to a DMI $|D| = 0.382 \pm 0.087$ mJ m^{-2} using $g = 2.13$ and $M_s = 0.88 \pm 0.16$ MA m^{-1}, with the same assumption as in section S1.1.2.b.

S1.2.3. Observation of AF coupled skyrmions in SAF2

Fig. S5a shows a STXM image of a track acquired at the Co edge and at zero external magnetic field, that displays a uniformly magnetised state. Fig. S5b shows the same track after the injection of multiple 10 ns current pulses with $J \approx 10^{12}$ A m^{-2}, leading to random nucleation of domains, most likely caused by the heating of the track. Fig. S5c and S5.d show two XMCD-STXM images of a skyrmion acquired respectively at the Co and Fe absorption edges in the area marked with a rectangle in Fig. S5b. It points out the AF coupling between FM1 and FM2 as explained previously, which is emphasised in the plot of the XMCD contrast in Fig. S5e (open symbols). Here, due to the larger size of the skyrmion, the signal, proportional to m_z, is best fitted by a 360° Bloch domain wall (DW) profile (solid lines). Both signals superimpose accurately, confirming the SAF character of the nucleated spin textures. Note that the effects observed in stray-field-coupled FM multilayers such as hybrid chiralities [12–14] are not expected is SAFs since the stray fields emanating from two adjacent FM layers compensate.

S1.2.4. Micromagnetic simulations of the current-induced skyrmion nucleation

To elaborate on the results of the current-induced nucleation/annihilation of skyrmions in SAF2, we perform numerical simulations. We first simulate a model system on COMSOL similar to the geometry used in the experiments (Fig. S6a). We assume that the entire SAF structure has a homogeneous metallic composition with resistivity 20 µΩ cm. The resistivity for the electrical

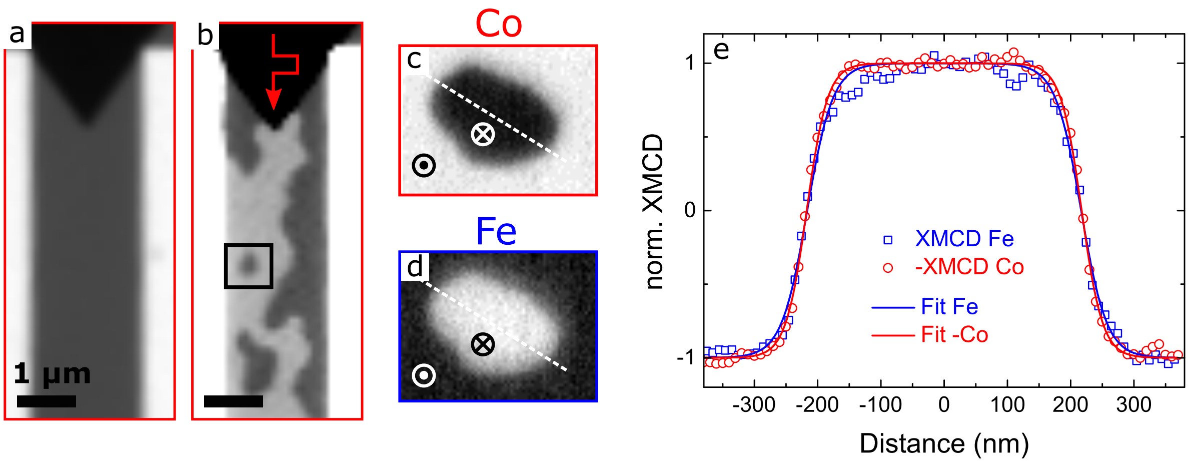

FIG. S5. **Observation of SAF skyrmions in SAF2.** **a** STXM image of a 2-μm-wide track at the Co edge. **b** Same image after injection of multiple 10 ns current pulses with amplitude $J \approx 10^{12}$ A m^{-2}. **c, d** XMCD-STXM images acquired at (**c**) the Co L_3 and (**d**) the Fe L_3 edge in the area of (**b**) delimited by a rectangle. **e** Normalised XMCD signal obtained from the line-scans along the white dashed lines in (**c**) and (**d**). The signal for Co is inverted. The solid lines are fit with a 360° Bloch DW profile. All images were acquired at room temperature and zero magnetic field.

contacts (Au) is 4 μΩ cm. The obtained current density is extracted on a finite difference grid and normalised with respect to its amplitude at a position far from the current injection region, J_0 (Fig. S6b). This normalised current density map is then used as an input mask for micromagnetic simulations in Mumax3.

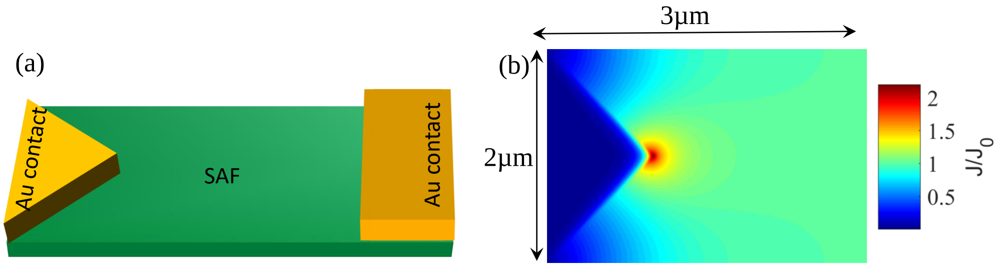

FIG. S6. **Current density simulations with COMSOL.** **a** Simulated geometry. **b** Current density map normalised with respect to the amplitude far from the injection region.

The micromagnetic simulations are performed with a single repetition of SAF (Fig. S4h, $N = 1$) with periodic boundary conditions along the z-axis to mimic the SAF2 multilayer ($N = 12$). The parameters used, however, are those measured in SAF2 and given in Table II. A slightly larger cell size of 2 nm × 2 nm × z nm is used to simulate the large sample size (3 μm × 3 μm × z nm). The simulation also contains randomly distributed grain with Gaussian variations in both K_u and

D (Full width at half maximum 5%). The anisotropy axis of the grains is also randomly allowed to deviate away from z-axis up to a maximum of 4°. The inter-grain exchange is also reduced by 10%. The simulations are performed at $T = 0$ K.

	Co/NiFe/Co	Co
t_{eff} (nm)	1.36	0.9
M_{s} (MA m^{-1})	0.9463	1.43
K_{u} (MJ m^{-3})	0.5948	1.6709
$\|D\|$ (mJ m^{-2})	0.382	1
A (pJ m^{-1})	6	16
α	0.45	0.3

TABLE II. **Micromagnetic parameters for SAF2.** Effective FM thickness, saturation magnetisation, uniaxial anisotropy constant, DMI constant, exchange constant and magnetic damping.

In Fig. S7a-d, we show the nucleation of a skyrmion using a current pulse of $J_0 = 15 \times 10^{12}$ A m^{-2} and pulse width of 0.4 ns. After the pulse, the generated texture is first allowed to evolve by integrating the LLG equation followed by an energy minimisation to establish the final stable state (Fig. S7d). We note here that the skyrmion in Fig. S7d is only stabilised due to the pinning provided by the randomly distributed grains as the parameters used for SAF2 do not allow the skyrmion to be a stable state. We also find that for our simulations, no nucleation can occur below a threshold current density of $J_0 = 12 \times 10^{12}$ A m^{-2}. This current density is around twenty times larger than the current density used in the experiments. We attribute this mismatch to the contribution of Joule heating in the nucleation process not considered in the simulation (performed at $T = 0$ K). At the tip region, where the current density is the largest, Joule heating can allow the nucleation process to begin at reduced threshold current densities. We have confirmed this reduction in threshold current from similar simulations performed at elevated temperatures. However, an exact quantitative picture for thermal contributions in a micromagnetic model is difficult to establish given the limitations of the Brown approach for inclusion of thermal field [15]. In Fig. S7e-h, we also show the annihilation of the skyrmion nucleated in Fig. S7d using the same current pulse, $J_0 = 15 \times 10^{12}$ A m^{-2}, but with opposite polarity. The pulse width is 0.15 ns after

which the skyrmion slowly shrinks and subsequently annihilates.

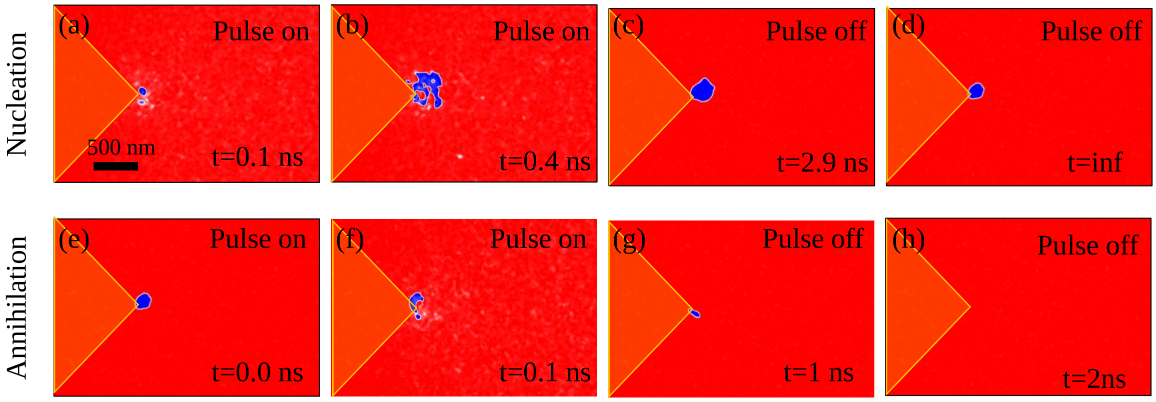

FIG. S7. **Micromagnetic simulations - Current-induced skyrmion nucleation/annihilation. a** Evolution of the magnetisation during skyrmion nucleation on application of current pulse with density $J_0 = 15 \times 10^{12}$ A m^{-2} and width 0.4 ns. **b** Skyrmion annihilation process with opposite polarity pulse with density $J_0 = 15 \times 10^{12}$ A m^{-2} and width 0.15 ns

S1.2.5. Current-induced nucleation of a SAF skyrmionium

The local current-induced nucleation in the SAF2 multilayer was studied by STXM. As shown in the main text, we have demonstrated the reproducible nucleation/annihilation of SAF skyrmions using current injection. We show in Fig. S8 that the successive injection of two positive pulses also allows us to nucleate *double skyrmion* spin textures similar to a skyrmionium.

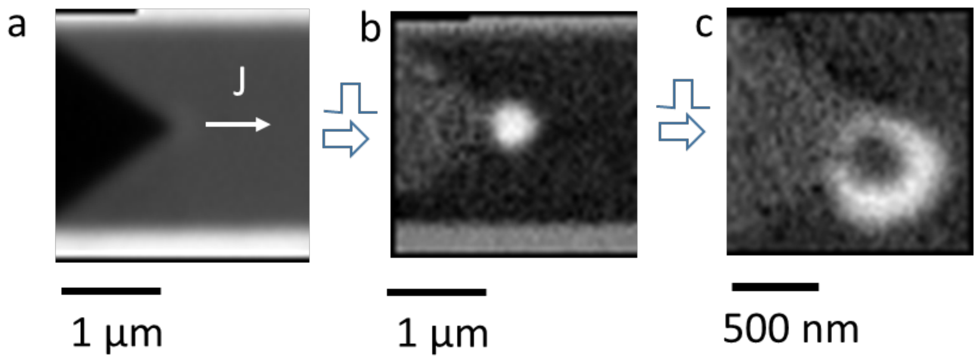

FIG. S8. **Nucleation of a skyrmionium spin texture. a** XMCD-STXM image (Co L_3 edge) of the SAF track with gold injector. **b** Same image but after the injection of a 5.3 ns current pulse with density 7×10^{11} A m^{-2} and **c** the subsequent injection of a 5.2 ns current pulse with density 5.6×10^{11} A m^{-2} .

S1.3. SAF3 multilayer: [Pt/Co/Ru]$_6$

S1.3.1. Material optimisation

The composition of the SAF3 multilayer used in the laser-induced skyrmion nucleation experiments (Fig. 5 in the main text) is the following: Ta(3)/Pt(2.25)/[Pt(0.75)/Co(1.49)/ Ru(0.85)]$_6$/ Pt(1.2) (thicknesses in nanometres). The samples were deposited by magnetron sputtering on Si(100) wafers as well as transparent MgO wafer substrates for the laser excitation. The Co layer was deposited a wedge so that the Co thickness of each layer was varied linearly between 1 and 1.8 nm along the 100 mm wafer. We show in Fig. S9a hysteresis loops measured in Ta(3)/Pt(3)/ Ru(0.85)/Pt(0.75)/Co(t_{Co})/Ru(0.85)/Ta(1.5), building block composing the SAF, at different positions on the wedge. It shows the spin reorientation transition around $t_{\mathrm{Co}} = 1.5$ nm. The hysteresis loops of the corresponding SAF structures are shown in Fig. S9b. As expected, a flat hysteresis loop with zero Kerr signal is observed at remanence, indicating that the SAF is compensated.

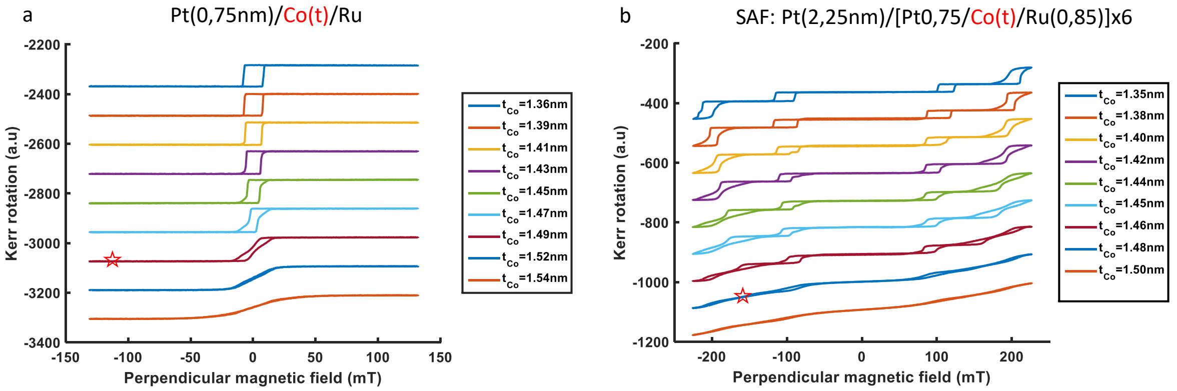

FIG. S9. **Optimisation of SAF3.** pMOKE OOP loops in **a** Ta(3)/Ru(0.85)/Pt(0.75)/Co(t)/Ru(0.85)/ Ta(1.5) and **b** Pt(2.25)/[Pt(0.75)/Co(t)/Ru(0.85]$_6$ measured at the same location on the wedge (curves are offset for clarity). The Co thickness varies between 1.33 nm and 1.55 nm. The red stars highlight the hysteresis loop of the sample where the laser-induced skyrmion nucleation experiments were performed.

S1.3.2. Measurement of the Dzyaloshinskii-Moriya interaction by Brillouin light scattering spectroscopy

The DMI was measured using BLS in a Ta(3)/Pt(3)/Ru(0.85)/Pt(0.75)/Co(1.5)/Ru(0.85)/ Pt(2) sample deposited by magnetron sputtering on a Si(100) substrate. Fig. S10a shows Δf as a function of the wave vector k. From a linear fit of Δf vs k, using $g = 2.21$ and $M_{\mathrm{s}} = 1.43 \pm 0.05$ MA m^{-1} (see section S1.1.2.a), we find $|D| = 0.77 \pm 0.04$ mJ m^{-2}.

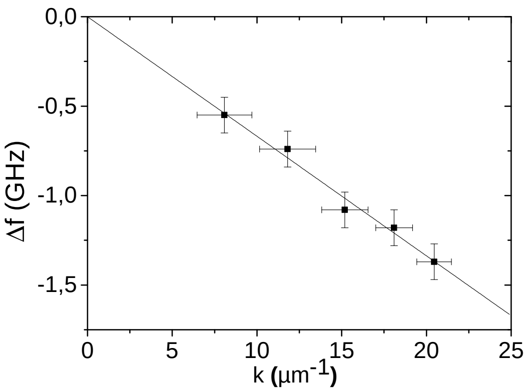

FIG. S10. **Brillouin light scattering measurements in SAF3.** Frequency shift as a function of the spin wave vector measured in Ta(3)/Pt(3)/Ru(0.85)/Pt(0.75)/Co(1.5)/Ru(0.85)/Pt(2) (thicknesses in nanometres). The error bars stand for the uncertainty due to the measurement resolution.

S1.3.3. Nucleation of SAF skyrmions using single fs laser pulse excitations

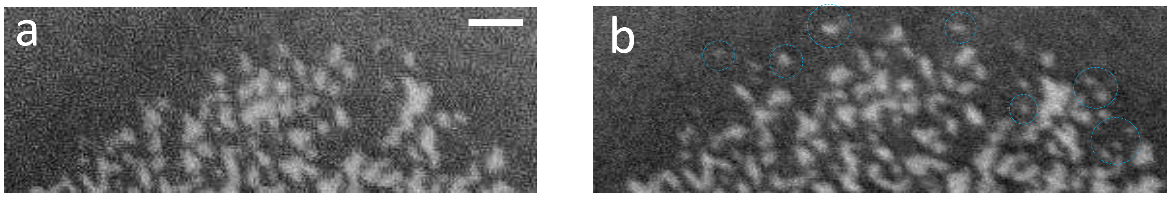

FIG. S11. **Nucleation of SAF skyrmions in SAF3 using a single fs laser pulse.** XMCD-PEEM images **a** before and **b** after the illumination of the sample by a single 80 fs laser pulse with fluence 36 ± 13 mJ cm^{-2}. The circles show the skyrmions nucleated by this process. Experiments are carried out at zero external magnetic field. Scale bar is 1 μm.

To observe the magnetic skyrmions and nucleate them, we used the XMCD-PEEM microscope at the UE49-PGMa beamline of the BessyII synchrotron in Berlin, Germany, featuring an in situ ultrafast pulsed laser source. The sample had a Co thickness of 1.49 nm, where the anisotropy is close to the spin reorientation transition (see Fig. S9a, red star) but still perpendicularly magnetised. We show in Fig. S11 XMCD-PEEM images of isolated worm domains and skyrmions before and after the illumination of the SAF sample with a single 80 fs laser pulse, leading to the nucleation of SAF skyrmions (marked by blue circles).

S1.3.4. Temperature model of femtosecond laser pulse excitation

To model the thermal excitation induced by the fs laser pulse on the SAF stack, we consider a 3-temperature model [16] that couples electron, phonon and spin temperature with a system of differential equations:

$$\begin{aligned} C_{\mathrm{e}}\frac{dT_{\mathrm{e}}}{dt} &= -G_{\mathrm{EL}}(T_{\mathrm{e}}-T_{\mathrm{l}}) - G_{\mathrm{ES}}(T_{\mathrm{e}}-T_{\mathrm{s}}) + P(t) \\ C_{\mathrm{l}}\frac{dT_{\mathrm{l}}}{dt} &= -G_{\mathrm{EL}}(T_{\mathrm{l}}-T_{\mathrm{e}}) - G_{\mathrm{SL}}(T_{\mathrm{l}}-T_{\mathrm{s}}) \\ C_{\mathrm{s}}\frac{dT_{\mathrm{s}}}{dt} &= -G_{\mathrm{ES}}(T_{\mathrm{s}}-T_{\mathrm{e}}) - G_{\mathrm{SL}}(T_{\mathrm{s}}-T_{\mathrm{l}}) \end{aligned}$$

Here T_{e}, T_{l} and T_{s} are respectively the electron, lattice, spin temperatures. $C_{\mathrm{e}} = \gamma T_{\mathrm{e}}$, C_{l} and C_{s} are the specific heat parameters of the electron, lattice, and spin. G_{EL}, G_{ES} and G_{SL} are the electron-lattice, electron-spin and spin-lattice thermal coupling parameters.

A	0.52
t_{eff} (nm)	18.54
γ (J m^{-3} K^{-2})	720
C_{l} (10^6 J m^{-3} K^{-1})	3
C_{s} (10^6 J m^{-3} K^{-1})	0.3
G_{EL} (10^{16} W m^{-3} K^{-1})	264
G_{ES} (10^{16} W m^{-3} K^{-1})	100
G_{SL} (10^{16} W m^{-3} K^{-1})	0.1
τ_{laser} (fs)	80

TABLE III. **3-temperature model parameters.**

The laser heat density in the layer ($P(t)$) is described in the model using the following equation:

$$P(t) = A\frac{F}{t_{\mathrm{eff}}\tau_{\mathrm{laser}}}\exp\left(-4\ln 2\frac{t^2}{\tau_{\mathrm{laser}}^2}\right) \tag{1}$$

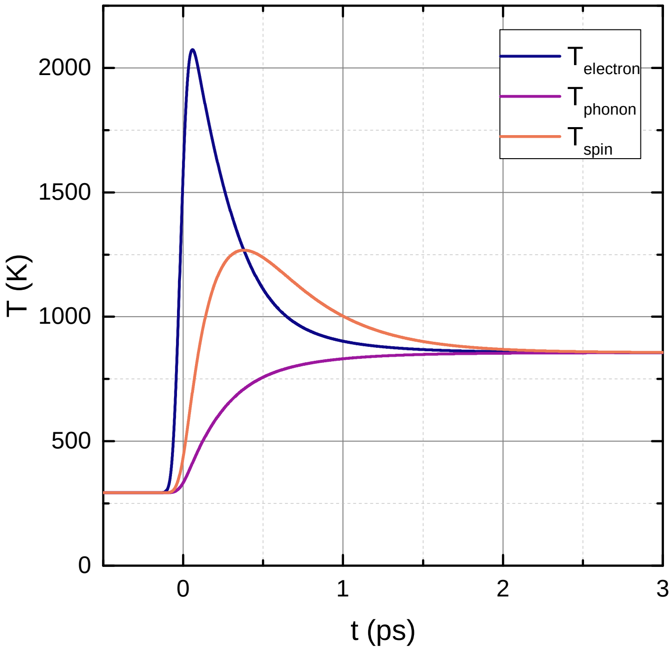

FIG. S12. **3-temperature model for laser excitation.** Evolution of the electron, lattice and spin temperature following a 80 fs laser pulse irradiation. The simulation time is shifted so that the peak of the laser illumination is at $t = 0$.

Here A is the absorbance of the magnetic multilayer, t_{eff} the thickness of the magnetic multilayer, F the laser fluence, and τ_{laser} the temporal beam width of 80 fs. For the simulations, we used the parameters of Ref [17, 18], which studied the ultra-fast demagnetisation of ultra-thin Pt/Co multilayers induced by laser pulses. These parameters are listed in Table III. The absorbance of the multilayer was calculated using the transfer matrix method described in Ref [19], taking into account the multilayer composition. A fluence of $F = 7$ mJ cm^{-2} is assumed corresponding to the one in the center of the beam of Fig. 5a in the main text.

The result of the model is shown in Fig. S12. The laser illumination leads to a fast increase of the spin temperature up to a maximum of 1268 K at 379 fs. This value is high compared to the Curie temperature expected in Pt/Co ultra-thin films (550-650 K) [20, 21] and close to the Curie temperature of bulk Co (1388 K). Thus, the laser pulse leads to an ultra-fast demagnetisation of the SAF stack. The spin temperatures relaxes back to a temperature of 856 K after about 2 ps, still above the expected Curie temperature of the stack. Such results is in line with previous time-resolved measurement of the demagnetisation process induced by ultra-short laser pulse in Pt/Co multilayers [21–23]. The longer time scale remagnetisation process is not included in the

model and depend on the thermal lattice diffusion. Time-resolved experiments show that it is typically in the tens of ps range for Pt/Co multilayers on Si substrates [22, 24] for similar fluence. For instance, a remagnetisation of Pt/Co multilayers of around 10 ps is measured by Shim et al. [22] after excitation by an ultra-short laser excitation at 800 nm wavelength.

S1.3.5. Micromagnetic simulation of the laser-induced skyrmion nucleation

Micromagnetic simulations were carried out to characterise the laser-induced nucleation of the SAF skyrmions. We used the parameters given in Table IV extracted from experiments corresponding to Fig. 3 in the main paper. The simulations include the 6 AF coupled Co layers. To mimic the demagnetisation induced by the laser pulse, the initial state is a random magnetisation configuration in a circular region (laser spot) in the center of the uniformly magnetised SAF structure. Note that the simulation window size is 1536 × 1536 nm^2 (cell size of 1 nm), smaller than the experimental laser beam (4.3 × 6.3 µm^2). Note also that the simulations are done at 0 K and therefore do not take into account the temperature dependence of the magnetic parameters.

t_{eff} (nm)	1.49
M_{s} (MA m^{-1})	1.43
K_{u} (MJ m^{-3})	1.311
$\lvert D \rvert$ (mJ m^{-2})	0.722
A (pJ m^{-1})	16

TABLE IV. **Micromagnetic parameters for SAF3.** Effective FM thickness, saturation magnetisation, uniaxial anisotropy constant, DMI constant and exchange constant.

Fig. S13 shows the magnetisation configuration at different times during the relaxation as obtained by solving the LLG equation (damping parameter of 0.3). At short time scales (Fig. S13b, $t = 0.05$ ns), small up and down domains connected by in-plane magnetised regions appear, which can be accounted for by the fast relaxation of the Heisenberg exchange energy. Larger domains with perpendicular magnetisation form rapidly after (Fig. S13c, $t = 0.2$ ns), which can be explained by the relaxation of the magnetic anisotropy energy. Small isolated skyrmions are also observed at this stage. The domains then increase in size and appear less dense (Fig. S13d, $t = 0.4$

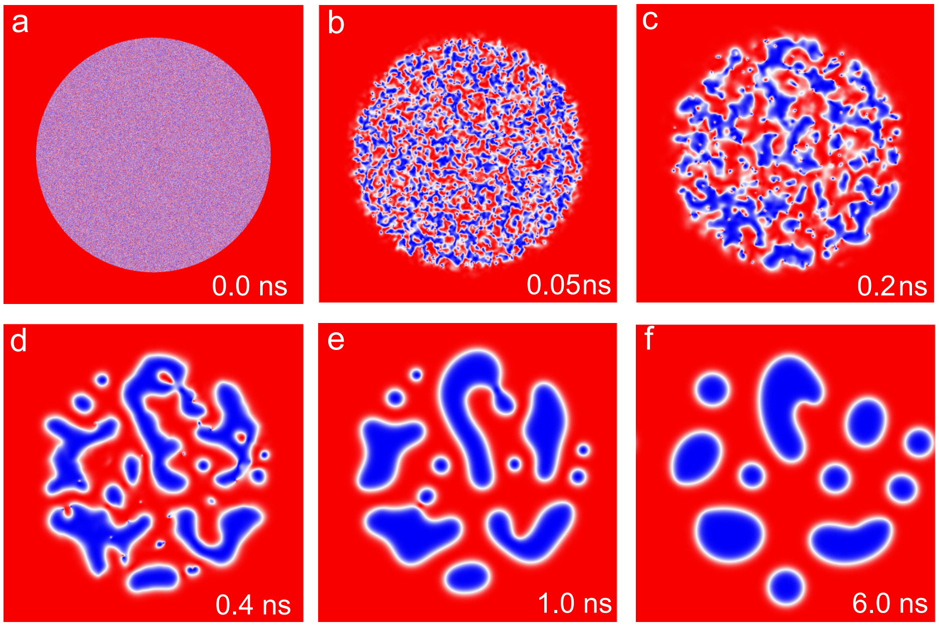

FIG. S13. **Micromagnetic simulations - Laser-induced SAF skyrmion nucleation.** Evolution of the magnetisation at different times (from **a** to **f**), starting from an initial circular demagnetised state mimicking the laser-induced demagnetisation (**a**). The simulation window size is 1536×1536 nm^2. The grid used is $1536 \times 1536 \times 12$ cells.

ns). While most of the smallest skyrmions turned out to be unstable and disappeared, the larger isolated skyrmions eventually stabilise at this stage. Their size slowly converges to the equilibrium value (Fig. S13e-f, $t = 1 - 6$ ns), which can be accounted for the relaxation of the DW energy. Note that the relaxation is slow after 0.4 ns and that due to the limited computation time (about 1 week), the final equilibrium state could not be reached. The latter was obtained by minimizing the micromagnetic energy using the steepest conjugate gradient method (function "minimize" in Mumax3 [10]) and is shown in Fig.5(d) of the main text. These results show that the typical time scale to relax to the equilibrium state is larger than 6 ns and likely of the order of a few tens of ns, in line with similar simulations for ferromagnetic skyrmions where a relaxation time scale of a few tens of ns was observed [25].

Finally note that although micromagnetic simulations capture most of the physics of the skyrmion formation after the laser-induced demagnetisation on a longer time scale, atomistic spin dynamics calculations would be needed to describe the laser-induced demagnetisation and the dynamics right after the laser pulse on the ps time scale.

S2. ADDITIONAL EXPERIMENTS: OBSERVATION OF CHIRAL DOMAIN WALLS AND SKYRMIONS IN A COMPENSATED SAF USING XMCD-PEEM

In STXM experiments, only the OOP component of the magnetisation is accessible. To determine the chirality of the observed spin textures, we performed XMCD-PEEM experiments on a compensated SAF bilayer deposited on a high-resistivity Si wafer. The SAF composition was Ta(3)/Pt(3)/Co(0.2)/$Ni_{80}Fe_{20}$(1)/Co(0.2)/Ru(0.85)/Pt(0.5)/Co(0.9)/Ru(0.85)/Ta(1.5) (thicknesses in nanometres), close to that of SAF1 and SAF2 where the AF alignment of the skyrmion magnetisation in each layer was observed. Fig. S14a shows the OOP hysteresis loop measured by VSM, which exhibits a very similar behaviour as the sample described in the previous section.

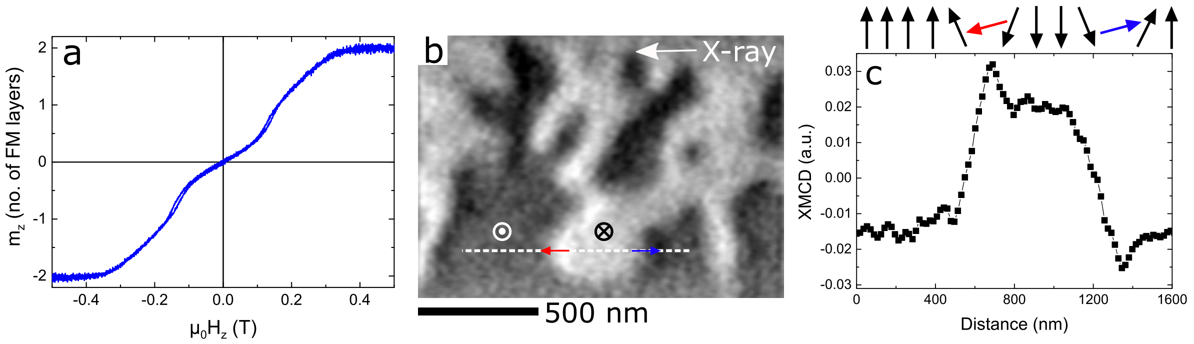

FIG. S14. **Chiral domain walls and skyrmions in a compensated SAF.** **a** OOP hysteresis loop measured by VSM on a compensated SAF with two constituent FM layers. **b** XMCD-PEEM image acquired at the Co L_3 absorption edge and at zero external magnetic field. **c** Line-scan of the XMCD contrast along the X-ray beam direction indicated by the white dashed line in (**b**).

Fig. S14b shows a XMCD-PEEM image acquired at the Co L_3 absorption edge and at zero external field. It displays alternate up/down domains as well as an isolated magnetic skyrmion of diameter $d_{sk} \approx 160$ nm. In Fig. S14c, we plot the XMCD contrast measured along the X-ray beam at the position marked with the white dashed line in Fig. S14b. Since the contrast is proportional to the projection of the X-ray beam direction on the magnetisation, we choose DWs perpendicular to the X-ray beam. The XMCD contrast exhibits the typical maximum and minimum corresponding respectively to a magnetisation parallel and anti-parallel to the beam [5]. Hence, the magnetisation rotates according to $\uparrow\leftarrow\downarrow\rightarrow\uparrow$, which is the signature of a left-handed Néel DW, consistent with the sign of the DMI measured by BLS in the different constituent FM layers. This strong white/black contrast is not observed for DWs perpendicular to the beam direction, which further supports that the DWs are of Néel type. Unfortunately, the NiFe layer could not be observed in this experiment

since the layer was buried deeper than the secondary electron escape length. Nevertheless, the AF coupling promotes DWs of the same chirality in both constituent FM layers so left-handed Néel spin textures are expected in the NiFe layer as well.

---

[1] Dieny, B., Gavigan, J. P. & Rebouillat, J. P. Magnetisation processes, hysteresis and finite-size effects in model multilayer systems of cubic or uniaxial anisotropy with antiferromagnetic coupling between adjacent ferromagnetic layers. *J. Phys.: Cond. Mat.* **2**, 159 (1990).

[2] Bloemen, P. J. H., van Kesteren, H. W., Swagten, H. J. M. & de Jonge, W. J. M. Oscillatory interlayer exchange coupling in Co/Ru multilayers and bilayers. *Phys. Rev. B* **50**, 13505 (1994).

[3] Belmeguenai, M. *et al.* Interfacial Dzyaloshinskii-Moriya interaction in perpendicularly magnetized Pt/Co/AlO_x ultrathin films measured by Brillouin light spectroscopy. *Phys. Rev. B* **91**, 180405(R) (2015).

[4] Nembach, H. T., Shaw, J. M., Weiler, M., Jué, E. & Silva, T. J. Linear relation between Heisenberg exchange and interfacial Dzyaloshinskii-Moriya interaction in metal films. *Nat. Phys.* **11**, 825–829 (2015).

[5] Boulle, O. *et al.* Room-temperature chiral magnetic skyrmions in ultrathin magnetic nanostructures. *Nat. Nanotech.* **11**, 449–454 (2016).

[6] Juge, R. *et al.* Current-Driven Skyrmion Dynamics and Drive-Dependent Skyrmion Hall Effect in an Ultrathin Film. *Phys. Rev. Appl.* **12**, 044007 (2019).

[7] Kuepferling, M. *et al.* Measuring interfacial Dzyaloshinskii-Moriya interaction in ultra thin films. *Preprint at arXiv:2009.11830 [cond-mat]* (2020).

[8] Bandiera, S., Sousa, R. C., Rodmacq, B. & Dieny, B. Asymmetric Interfacial Perpendicular Magnetic Anisotropy in Pt/Co/Pt Trilayers. *IEEE Mag. Lett.* **2**, 3000504 (2011).

[9] Thiaville, A., Rohart, S., Jué, É., Cros, V. & Fert, A. Dynamics of Dzyaloshinskii domain walls in ultrathin magnetic films. *Europhys. Lett.* **100**, 57002 (2012).

[10] Vansteenkiste, A. *et al.* The design and verification of MuMax3. *AIP Adv.* **4**, 107133 (2014).

[11] Hellwig, O., Berger, A., Kortright, J. B. & Fullerton, E. E. Domain structure and magnetization reversal of antiferromagnetically coupled perpendicular anisotropy films. *J. Magn. Magn. Mater.* **319**, 13–55 (2007).

[12] Legrand, W. *et al.* Hybrid chiral domain walls and skyrmions in magnetic multilayers. *Sci. Adv.* **4**, eaat0415 (2018).

[13] Dovzhenko, Y. *et al.* Magnetostatic twists in room-temperature skyrmions explored by nitrogen-vacancy center spin texture reconstruction. *Nat. Comm.* **9**, 2712 (2018).

[14] Li, W. *et al.* Anatomy of Skyrmionic Textures in Magnetic Multilayers. *Adv. Mater.* **31**, 1807683 (2019).

[15] Brown, W. F. Thermal Fluctuations of a Single-Domain Particle. *Phys. Rev.* **130**, 1677–1686 (1963).

[16] Beaurepaire, E., Merle, J.-C., Daunois, A. & Bigot, J.-Y. Ultrafast Spin Dynamics in Ferromagnetic Nickel. *Phys. Rev. Lett.* **76**, 4250–4253 (1996).

[17] Kichin, G. *et al.* From Multiple- to Single-Pulse All-Optical Helicity-Dependent Switching in Ferromagnetic Co/Pt Multilayers. *Phys. Rev. Appl.* **12**, 024019 (2019).

[18] Bergeard, N. *et al.* Hot-Electron-Induced Ultrafast Demagnetization in Co/Pt Multilayers. *Phys. Rev. Lett.* **117**, 147203 (2016).

[19] Byrnes, S. J. Multilayer optical calculations. *Preprint at arXiv:1603.02720 [physics.comp-ph]* (2020).

[20] van Kesteren, H. W. & Zeper, W. B. Controlling the Curie temperature of Co/Pt multilayer magneto-optical recording media. *J. Magn. Magn. Mater.* **120**, 271–273 (1993).

[21] Kuiper, K. C. *et al.* Spin-orbit enhanced demagnetization rate in Co/Pt-multilayers. *Appl. Phys. Lett.* **105**, 202402 (2014).

[22] Shim, J.-H. *et al.* Ultrafast dynamics of exchange stiffness in Co/Pt multilayer. *Comm. Phys.* **3**, 1–8 (2020).

[23] von Korff Schmising, C. *et al.* Imaging Ultrafast Demagnetization Dynamics after a Spatially Localized Optical Excitation. *Phys. Rev. Lett.* **112**, 217203 (2014).

[24] Vaskivskyi, I. *et al.* Element-Specific Magnetization Dynamics in Co–Pt Alloys Induced by Strong Optical Excitation. *J. Phys. Chem. C* **125**, 11714–11721 (2021).

[25] Je, S.-G. *et al.* Creation of Magnetic Skyrmion Bubble Lattices by Ultrafast Laser in Ultrathin Films. *Nano Lett.* **18**, 7362–7371 (2018).
